# Utility of Cardiac CT for Cardiomyopathy Phenotyping

**DOI:** 10.3390/tomography11030039

**Published:** 2025-03-20

**Authors:** Ramzi Ibrahim, Mahmoud Abdelnabi, Girish Pathangey, Juan Farina, Steven J. Lester, Chadi Ayoub, Said Alsidawi, Balaji K. Tamarappoo, Clinton Jokerst, Reza Arsanjani

**Affiliations:** 1 Department of Cardiovascular Medicine, Mayo Clinic, Phoenix, AZ 85054, USA; ibrahim.ramzi@mayo.edu (R.I.); abdelnabi.mahmoud@mayo.edu (M.A.); pathangey.girish@mayo.edu (G.P.); farina.juanmaria@mayo.edu (J.F.); lester.steven@mayo.edu (S.J.L.); ayoub.chadi@mayo.edu (C.A.); alsidawi.said@mayo.edu (S.A.); tamarappoo.balaji@mayo.edu (B.K.T.); 2 Department of Radiology, Mayo Clinic, Scottsdale, AZ 85054, USA; jokerst.clinton@mayo.edu

**Keywords:** CT, cardiomyopathy, cardiovascular

## Abstract

Cardiac computed tomography (CT) has rapidly advanced, becoming an invaluable tool for diagnosing and prognosticating various cardiovascular diseases. While echocardiography and cardiac magnetic resonance imaging (CMR) remain the gold standards for myocardial assessment, modern CT technologies offer enhanced spatial resolution, making it an essential tool in clinical practice. Cardiac CT has expanded beyond coronary artery disease evaluation, now playing a key role in assessing cardiomyopathies and structural heart diseases. Innovations like photon-counting CT enable precise estimation of myocardial extracellular volume, facilitating the detection of infiltrative disorders and myocardial fibrosis. Additionally, CT-based myocardial strain analysis allows for the classification of impaired myocardial contractility, while quantifying cardiac volumes and function remains crucial in cardiomyopathy evaluation. This review explores the emerging role of cardiac CT in cardiomyopathy phenotyping, emphasizing recent technological advancements.

## 1. Introduction

Cardiac computed tomography (CT) scans have advanced significantly in recent years, providing both diagnostic and prognostic value across a wide range of cardiovascular diseases. With high spatial resolution, CT enables detailed plaque characterization, including the identification of high-risk features [1]. Beyond evaluating atherosclerotic coronary plaque and stenosis, cardiac CT has demonstrated strong prognostic capabilities for assessing myocardial infarction risk [2,3,4]. Functional tools, such as CT-derived fractional flow reserve (FFRct) and perfusion analysis, have further enhanced its utility in clinical practice [4]. Additionally, cardiac CT plays a crucial role in pre-procedural planning for minimally invasive interventions, including transcatheter valve replacement, left atrial appendage occlusion, lead extraction, and evaluation of valvular pathology [5,6].

Although cardiac magnetic resonance imaging (CMR) and echocardiography remain gold standards for myocardial assessment [7], cardiac CT has emerged as a widely accessible alternative with faster acquisition times and compatibility with cardiac devices [7]. For example, while CMR often requires prolonged imaging sessions, which may be challenging for some patients, cardiac CT provides rapid, non-invasive information. Its spatial resolution (sub-mm), compared to CMR and volumetric data acquisition, enables high-fidelity multiplanar reconstructions that can be manipulated post-scan.

Cardiac CT is also valuable, though less commonly used, for phenotyping structural heart diseases and cardiomyopathies. In such cases, when coronary imaging is not the primary focus, patient preparation is less intensive, with a reduced need for heart rate control or pre-medication with nitroglycerine. This is due to the enhanced contrast resolution and spatial definition afforded by advanced CT technologies. These advancements support applications such as estimating myocardial extracellular volume, myocardial strain, volumetry, and morphology.

This review highlights the evolving role of cardiac CT in cardiomyopathy phenotyping and explores the latest advancements in CT-based techniques.

## 2. Myocardial Morphology and Function

Cardiac CT is a valuable tool for evaluating myocardial function and morphology, particularly with protocols that use retrospective ECG gating. This technique captures images throughout the entire cardiac cycle, combining the excellent spatial resolution of CT with the ability to assess dynamic cardiac function. However, capturing images across the full cardiac cycle requires a significantly higher radiation dose. Compared to MRI, CT demonstrates similar accuracy in assessing wall motion abnormalities and one study even demonstrated that cardiac CT outperformed two-dimensional echocardiography in reproducibility and accuracy [8]. Despite early studies indicating an overestimation of ventricular volumes with CT, advancements in technology have significantly improved its accuracy [9,10,11,12,13].

In addition to being less widely accessible than echocardiography, cardiac CT requires the use of radiographic contrast to clearly define both the right and left ventricular endocardial borders, ensuring balanced contrast opacification. In contrast, neither echocardiography nor CMR requires a contrast agent for endocardial border definition.

**Figure 1:**
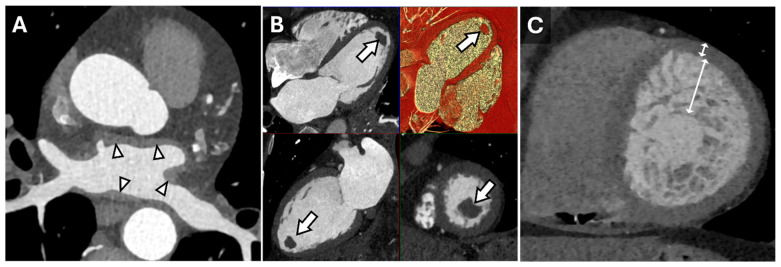
Cardiac Findings and CT Imaging. Axial reconstruction from a contrast-enhanced cardiac CT (**A**) demonstrating irregular left atrial wall thickening (arrowheads) reflecting amyloid deposition in the setting of cardiac amyloidosis. Multiplanar (HLA, VLA, SAX, 3D) diastolic images (**B**) from a contrast-enhanced cardiac CT in a patient with a left ventricular apical thrombus (arrows). Short-axis end-diastolic image from a contrast-enhanced cardiac CT in a patient with myocardial non-compaction (**C**) demonstrating very prominent trabeculation of the mid-apical left ventricle with an increased ratio of non-compacted to compacted myocardium measuring >> 2.3:1 (double-headed arrows).

**Figure 2:**
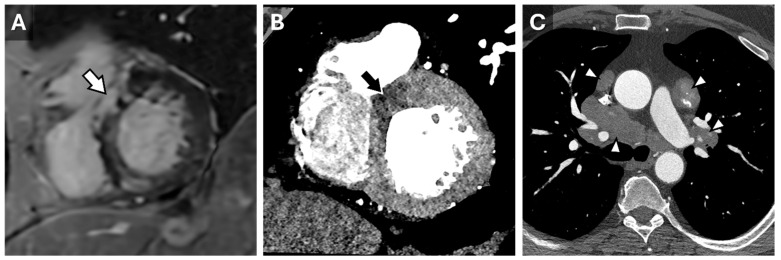
Sarcoidosis in CT Cardiac Imaging. CMR short-axis delayed-enhanced inversion recovery image through the LV base (**A**) from a patient with complete heart block and sarcoidosis with myocardial involvement. There is nonvascular delayed enhancement in the basilar septum (arrow). A contrast-enhanced cardiac CT from the same patient demonstrates a correlating area of decreased myocardial perfusion ((**B**), arrow). Axial reconstruction from the same CT (**C**) demonstrates partially calcified mediastinal and hilar lymphadenopathy (arrowheads) consistent with adenopathy in the setting of sarcoidosis.

Cardiac CT is well-suited for the evaluation of morphologic features, such as wall thickness and chamber dimensions, given its excellent spatial resolution and volumetric acquisition. It is worth noting that most CT coronary angiography is performed during end-systole or mid-diastole, which may result in an overestimation of wall thickness and underestimate end-diastolic volumes [14]. Additional features, such as atrial wall thickening, left ventricular thrombus, or non-compaction, can be identified regardless of the cardiac cycle phase (Figure 1) [15]. Thrombus detection, in particular, is often more reliable with CT than echocardiography, as it appears as a filling defect in a contrast-filled chamber. Beyond cardiac morphology, CT can reveal extracardiac findings that may provide broader diagnostic insights. For instance, bilateral hilar adenopathy, visible on cardiac CT, may suggest sarcoidosis, expanding the diagnostic perspective beyond cardiac pathology (Figure 2).

## 3. Myocardial Strain

Myocardial strain measures the extent of myocardial deformation, encompassing longitudinal, circumferential, radial, and torsional components [16]. It is a valuable tool for assessing myocardial contractility, often detecting impairment before left ventricular ejection fraction (LVEF) declines [17,18]. Strain offers prognostic and diagnostic insights beyond LVEF, with studies showing that it is superior in predicting major adverse cardiac events (MACEs); however, most studies have used echocardiography or CMR-derived strain patterns [17,19,20]. Stain is also a reliable marker for predicting cardiotoxicity in chemotherapy patients and possibly for the timing of surgery in asymptomatic valve disease [18,21].

**Figure 3:**
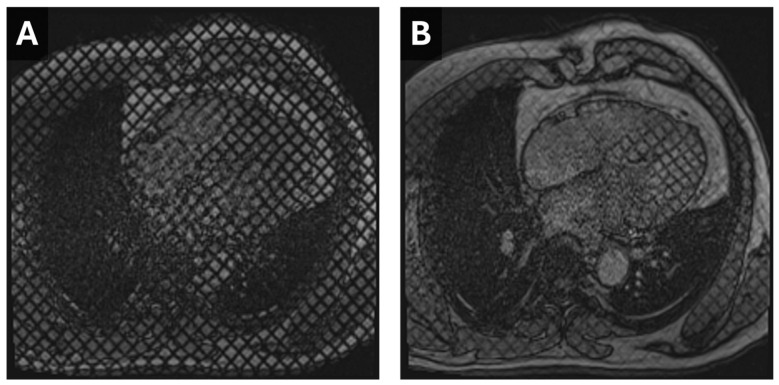
Cardiac MR Strain Evaluation. Static horizontal long-axis diastolic (**A**) and systolic (**B**) grid-tagged images from a cardiac MRI. Deformation of the grid as the myocardium contracts can be used to estimate strain.

**Figure 4:**
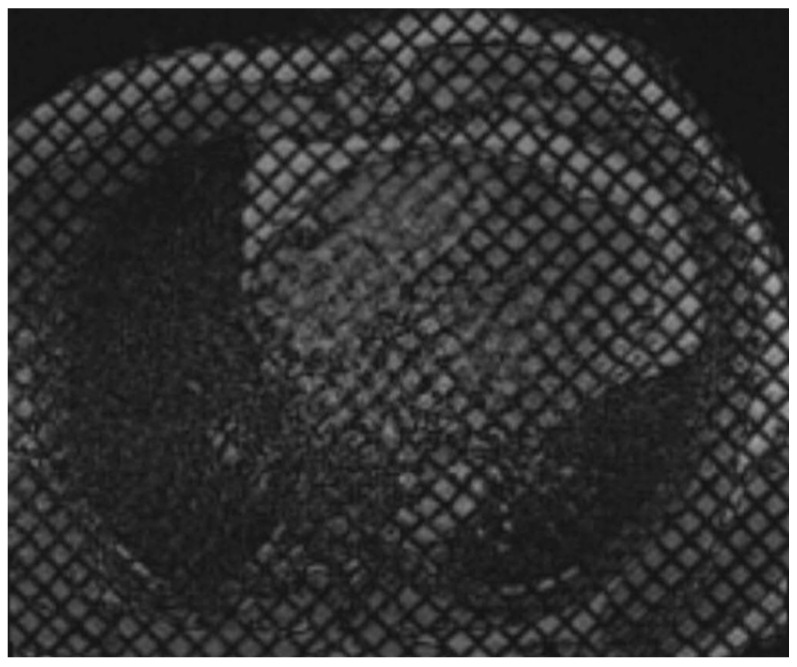
Cardiac MR Strain Evaluation. Cine horizontal long-axis grid-tagged images from a cardiac MRI. Deformation of the grid as the myocardium contracts can be used to estimate strain.

Traditionally measured via echocardiography or CMR, modern cardiac CT software can now estimate myocardial strain by analyzing pixel features, anatomic landmarks, and endocardial and epicardial borders (Figure 3 and Figure 4). However, CT-derived strain is limited by a lower temporal resolution, which may result in a weaker correlation to either MRI or Echo-derived strain values, particularly radial and circumferential measures. It is noted that, currently, neither radial nor circumferential values derived by either MRI or Echo are used in routine clinical practice due to high variability and low reproducibility; thus, this limitation with CT is currently more intellectual than clinical. In valvular disease, CT-derived strain has shown a moderate correlation with echocardiography (r = 0.6, *p* < 0.001), though it tends to underestimate strain values [22]. Despite this, CT provides a feasible method for strain assessment in advanced valve disease, complementing echocardiographic findings.

Strain patterns also correlate with coronary artery calcium (CAC) scores and stenosis severity. In a study of CAD-RADS groups, a standardized classification system used to estimate the severity of coronary artery disease on CT imaging, higher CAC and stenosis levels were associated with reduced global strain across all directions, likely reflecting diminished coronary blood flow [23]. These studies suggest the utility of CT-derived strain in evaluating coronary artery disease, especially when LVEF and chamber volumes remain normal.

Although data on reference ranges for strain measurements using CT imaging remain limited, a 2024 established baseline strain values of the four cardiac chambers in healthy adults, accounting for sex and age differences [23]. Women demonstrated higher global longitudinal, circumferential, and radial strain values compared to men, underscoring the need to consider sex-specific differences in clinical practice. These data provide a foundation for integrating CT strain into routine cardiac assessment.

## 4. Myocardial ECV

Traditionally, CMR has been the primary method for measuring myocardial extracellular volume (ECV) measurements. However, CT-guided ECV has proven effective in identifying diffuse myocardial fibrosis and signs of infiltrative cardiomyopathy and myocarditis, offering important prognostic insights for both ischemic and non-ischemic cardiomyopathies [24,25]. Modern CT software platforms can generate semi-automated ECV maps from non-contrast and contrast-enhanced images, and dual-energy CT techniques offer enhanced image quality, reduced artifacts, and lower levels of radiation exposure [26,27]). Fully automated models are also being developed to enhance image resolution and reduce manual editing in ECV measurement [28,29].

ECV estimation requires a dedicated cardiac CT acquisition protocol. While coronary CT imaging can assess myocardial thickness and fat infiltration, ECV quantification requires a broader imaging focus. The first human study exploring this approach was conducted in 2005, with 28 post-myocardial infarction patients, using a 16-slice cardiac CT, showing that CT-based fibrosis assessment was concordant with CMR findings [30]. A 2008 study with 71 patients further confirmed that cardiac CT can reliably identify myocardial fibrosis [30].

**Figure 5:**
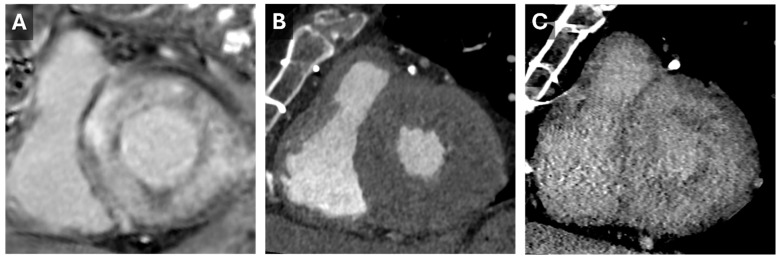
Cardiac Amyloid Findings. CMR short-axis delayed-enhanced inversion recovery image through the LV base (**A**) from a patient with severe cardiac amyloidosis with diffuse transmural delayed enhancement. Short axis images through the LV base from a previous cardiac CT with arterial (**B**) and 5 min delayed (**C**) phases. The delayed phase demonstrates diffuse nonvascular enhancement that correlates well with the findings seen on the subsequent CMR.

Current protocols involve both non-contrast and contrast-enhanced acquisitions, followed by a delayed phase approximately 5–10 min later [31]. The contrast volume used is often at least 1.5 mL/kg, which is higher than for coronary assessments. Iodinated contrast behaves similarly to gadolinium accumulating in abnormal myocardium with greater extracellular space, such as in areas of diffuse fibrosis or amyloid deposition (Figure 5), which allows for more accurate disease detection.

**Figure 6:**
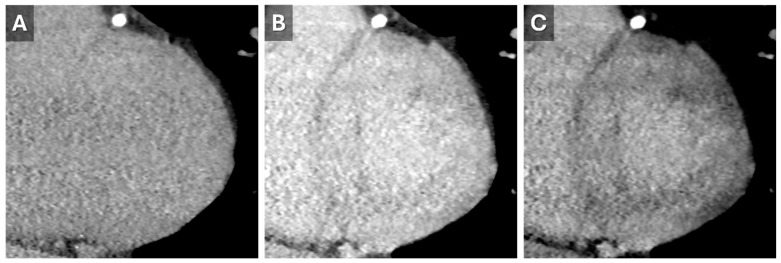
Dual-energy Cardiac CT Imaging. Short-axis reconstructions through the LV base from a 5 min delayed phase of a contrast-enhanced cardiac CT that utilized spectral (dual energy) technique. Images from the high-energy (**A**) and low-energy (**B**) datasets show the increase in iodine conspicuity from the low-energy data (**B**). A virtual monoenergetic 50 keV reconstruction (**C**) demonstrates a good mix of anatomic information and contrast conspicuity.

Despite its advantages, CT for ECV measurement has limitations, including a low signal-to-noise ratio and the need for higher contrast doses. Dual-energy CT can address some of these issues by reducing contrast requirements and utilizing varying tube potentials (Figure 6) [26]. This approach was validated in 2020 when Ohta et al. demonstrated a significant correlation between CT- and CMR-based ECV quantification in 23 patients [32].

For example, one study evaluated the impact of ECV calculation on clinical outcomes in 70 patients with dilated cardiomyopathy [33]. Elevated ECV, particularly above 32.26%, was associated with higher rates of MACE based on receiver operating curves. This finding held true as the only independent predictor of MACE in the multivariate Cox proportional hazards model, as compared to other variables.

## 5. Ischemic Evaluation

Distinguishing between ischemic and non-ischemic causes of dilated cardiomyopathies is essential for guiding management and prognosis. Identifying ischemia as the underlying cause can significantly impact treatment decisions, particularly regarding revascularization. Cardiac CT offers an accurate, non-invasive alternative to invasive coronary angiography, providing anatomical detail and identifying potential targets for intervention [34].

Cardiac CT may be used to evaluate coronary artery disease in those with a reduced LVEF due to its high negative predictive value [35]. During non-contrast-enhanced CT assessments for coronary disease, the presence of calcified coronary arteries was largely used to infer the likelihood of significant coronary artery disease [36]. Advancements in CT imaging with contemporary CADRADS-2.0 guidelines have enhanced the utility of CT coronary evaluation, particularly through stenosis quantification and high-risk plaque feature identification [37].

CT imaging is also useful for assessing myocardial fibrosis and post-myocardial infarction changes, such as scar tissue and fat deposition. Fat in the myocardium typically appears in a subendocardial distribution within the affected coronary artery territory, providing additional diagnostic value [38,39]. In addition, hypoattenuation, often observed in patients with CAD, can be effectively assessed using CT-based perfusion imaging, which may be even more sensitive than radionuclide myocardial perfusion imaging [38,39]. However, this method requires longer radiation exposure and contrast (Table 1). A notable limitation of CT perfusion imaging is its inability to evaluate the reversibility of perfusion defects, a capability provided by radionuclide imaging.

## 6. Hypertrophic Cardiomyopathy

Cardiac CT is also effective for assessing hypertrophic cardiomyopathy (HCM), including asymmetrical ventricular hypertrophy as well as related findings such as systolic anterior motion of the mitral valve or the presence of an apical pouch in those with the apical variant [40,41]. Patients with hypertrophic cardiomyopathy (HCM) may present with chest pain, which can be challenging to attribute solely to the cardiomyopathy or to potential concomitant coronary artery disease (CAD) [41]. Both conditions can cause similar symptoms due to impaired blood flow or increased myocardial oxygen demand. Cardiac CT scanning provides a valuable tool for differentiation by allowing for the precise evaluation of coronary anatomy to rule out significant CAD while also assessing myocardial hypertrophy, fibrosis, and other structural abnormalities characteristic of HCM. This comprehensive assessment aids in accurate diagnosis and targeted management [41].

### Restrictive Cardiomyopathy

Restrictive cardiomyopathy (RCM) is a condition characterized by stiff ventricular walls leading to impaired diastolic function and restrictive filling (Jan). The left ventricle (LV) typically retains normal or near-normal dimensions, while biatrial enlargement is a common feature due to chronically elevated filling pressures [42]. Most cases arise from underlying infiltrative, storage, or fibrotic diseases, though some remain idiopathic. Cardiac CT provides detailed structural assessment and tissue characterization in RCM, complementing traditional imaging modalities such as echocardiography and CMR. ECV mapping with CT has proven useful in detecting myocardial infiltration and fibrosis, particularly in cardiac amyloidosis, where elevated ECV values correlate with disease burden [37]. Similarly, dual-energy CT can help differentiate iron overload cardiomyopathy (e.g., hemochromatosis) by identifying iron deposits that appear as low-attenuation areas on spectral imaging [43]. In addition to quantifying myocardial fibrosis, CT is effective for detecting structural abnormalities associated with RCM, such as endomyocardial fibrosis, intracardiac thrombi, and myocardial calcifications [15]. In lysosomal storage disorders, like Anderson–Fabry disease, CT may reveal distinctive patterns of left ventricular hypertrophy, particularly involving the basal inferolateral wall, which can aid in early diagnosis [44]. As these technologies continue to evolve, CT will likely become an increasingly important tool for noninvasive myocardial assessment in restrictive cardiomyopathies.

## 7. Arrhythmogenic Cardiomyopathy

Replacement of the myocardial tissue with fibrofatty tissue is a hallmark finding in arrhythmogenic right ventricular cardiomyopathy (ARVC). Although standardized diagnostic cut-offs for ARVC based on right ventricular morphology are limited, cardiac CT plays a critical role in identifying key structural abnormalities, such as right ventricular dilation, systolic dysfunction, and focal bulging. These imaging findings, combined with clinical parameters and electrocardiographic features, provide a more comprehensive assessment of the disorder.

Recent research has demonstrated a positive correlation between invasive electro-anatomical mapping and right ventricular tissue heterogeneity detected by cardiac CT further supporting its utility in diagnosing ARVC [45]. However, it is important to note that cardiac CT may occasionally overestimate right ventricular diastolic volume, which may result from variations in respiratory cycle phases or contrast administration during imaging [46]. Despite these limitations, cardiac CT remains an invaluable tool for the evaluation and diagnosis of ARVC.

## 8. Cardiac Ablation Pre-Procedural Planning

Atrial and ventricular arrhythmias are common in patients with cardiomyopathy, particularly those with ischemic etiologies. Using CMR imaging to identify arrhythmic substrates has been shown to improve periprocedural outcomes [47]. However, challenges such as long acquisition times, possible unstable arrhythmias, or interference from implanted cardiac devices can limit CMR use. In contrast, cardiac CT has become valuable for preprocedural planning in ventricular arrhythmia ablation [48]. One study explored the idea that cardiac CT combined with invasive electrophysiological mapping helps to identify anatomical substrates. Additionally, cardiac CT can assess coronary artery disease and myocardial fibrosis, providing a comprehensive evaluation during electrophysiological procedures.

## 9. Conclusions

Cardiac CT offers valuable insights into phenotyping undifferentiated cardiomyopathy, complementing echocardiography and CMR. While echocardiography and CMR are more commonly used to assess ventricular function and tissue characterization, cardiac CT provides detailed information on myocardial morphology, function, strain patterns, fibrosis, infiltrative diseases, and peri-operative planning. When appropriate, cardiac CT may play an expanding role in cardiomyopathy phenotyping algorithms.

## Figures and Tables

**Table 1:** Myocardial Hypoperfusion in CT Cardiac Imaging. Comparison of CT cardiac imaging modalities versus radionuclide myocardial perfusion imaging.

Assessment Method	Key Features	Advantages	Limitations	Additional Insights
CT-Based Perfusion Imaging	Identifies areas of hypoattenuation.	More sensitive than radionuclide imaging. - Provides stress and rest images.	Requires longer radiation times and contrast. - Cannot determine the reversibility of perfusion defects.	- CT perfusion defects present in both stress and rest images, similar to radionuclide studies. - Uncertain for viability assessment.
CT for Post-MI Scar Assessment	- Differentiates fat from myocardium. - Identifies fibrous scar tissue and fat deposition post-MI (6 months).	Offers additional diagnostic insights through fat detection.	- Primarily detects late-stage myocardial changes. - Limited for early post-MI assessment.	- Fat in myocardium appears in subendocardial distribution within the affected coronary artery territory.
Radionuclide Myocardial Perfusion Imaging	Assesses myocardial perfusion and determines reversibility of defects.	Can determine reversibility of perfusion defects.	Less sensitive compared to CT-based imaging.	Provides comprehensive viability assessment.

## References

[1] Clayton B., Roobottom C., Morgan-Hughes G. (2014). Assessment of the myocardium with cardiac computed tomography. Eur. Heart J. Cardiovasc. Imaging.

[2] Gulati M., Levy P.D., Mukherjee D., Amsterdam E., Bhatt D.L., Birtcher K.K., Blankstein R., Boyd J., Bullock-Palmer R.P., Conejo T. (2021). 2021 AHA/ACC/ASE/CHEST/SAEM/SCCT/SCMR Guideline for the Evaluation and Diagnosis of Chest Pain: A Report of the American College of Cardiology/American Heart Association Joint Committee on Clinical Practice Guidelines. Circulation.

[3] Investigators S.-H., Newby D.E., Adamson P.D., Berry C., Boon N.A., Dweck M.R., Flather M., Forbes J., Hunter A., Lewis S. (2018). Coronary CT Angiography and 5-Year Risk of Myocardial Infarction. N. Engl. J. Med..

[4] Fairbairn T.A., Nieman K., Akasaka T., Norgaard B.L., Berman D.S., Raff G., Hurwitz-Koweek L.M., Pontone G., Kawasaki T., Sand N.P. (2018). Real-world clinical utility and impact on clinical decision-making of coronary computed tomography angiography-derived fractional flow reserve: Lessons from the ADVANCE Registry. Eur. Heart J..

[5] Pulerwitz T.C., Khalique O.K., Leb J., Hahn R.T., Nazif T.M., Leon M.B., George I., Vahl T.P., D’Souza B., Bapat V.N. (2020). Optimizing Cardiac CT Protocols for Comprehensive Acquisition Prior to Percutaneous MV and TV Repair/Replacement. JACC Cardiovasc. Imaging.

[6] Korsholm K., Berti S., Iriart X., Saw J., Wang D.D., Cochet H., Chow D., Clemente A., De Backer O., Moller Jensen J. (2020). Expert Recommendations on Cardiac Computed Tomography for Planning Transcatheter Left Atrial Appendage Occlusion. JACC Cardiovasc. Interv..

[7] Hendel R.C., Patel M.R., Kramer C.M., Poon M., Hendel R.C., Carr J.C., Gerstad N.A., Gillam L.D., Hodgson J.M., Kim R.J. (2006). ACCF/ACR/SCCT/SCMR/ASNC/NASCI/SCAI/SIR 2006 appropriateness criteria for cardiac computed tomography and cardiac magnetic resonance imaging: A report of the American College of Cardiology Foundation Quality Strategic Directions Committee Appropriateness Criteria Working Group, American College of Radiology, Society of Cardiovascular Computed Tomography, Society for Cardiovascular Magnetic Resonance, American Society of Nuclear Cardiology, North American Society for Cardiac Imaging, Society for Cardiovascular Angiography and Interventions, and Society of Interventional Radiology. J. Am. Coll. Cardiol..

[8] Yamamuro M., Tadamura E., Kubo S., Toyoda H., Nishina T., Ohba M., Hosokawa R., Kimura T., Tamaki N., Komeda M. (2005). Cardiac functional analysis with multi-detector row CT and segmental reconstruction algorithm: Comparison with echocardiography, SPECT, and MR imaging. Radiology.

[9] Brodoefel H., Reimann A., Klumpp B., Fenchel M., Heuschmid M., Burgstahler C., Schroeder S., Miller S., Claussen C.D., Scheule A.M. (2007). Sixty-four-slice CT in the assessment of global and regional left ventricular function: Comparison with MRI in a porcine model of acute and subacute myocardial infarction. Eur. Radiol..

[10] Busch S., Johnson T.R., Wintersperger B.J., Minaifar N., Bhargava A., Rist C., Reiser M.F., Becker C., Nikolaou K. (2008). Quantitative assessment of left ventricular function with dual-source CT in comparison to cardiac magnetic resonance imaging: Initial findings. Eur. Radiol..

[11] Krishnam M.S., Tomasian A., Iv M., Ruehm S.G., Saleh R., Panknin C., Goldin J.G. (2008). Left ventricular ejection fraction using 64-slice CT coronary angiography and new evaluation software: Initial experience. Br. J. Radiol..

[12] Heuschmid M., Rothfuss J.K., Schroeder S., Fenchel M., Stauder N., Burgstahler C., Franow A., Kuzo R.S., Kuettner A., Miller S. (2006). Assessment of left ventricular myocardial function using 16-slice multidetector-row computed tomography: Comparison with magnetic resonance imaging and echocardiography. Eur. Radiol..

[13] Plumhans C., Muhlenbruch G., Rapaee A., Sim K.H., Seyfarth T., Gunther R.W., Mahnken A.H. (2008). Assessment of global right ventricular function on 64-MDCT compared with MRI. AJR Am. J. Roentgenol..

[14] Gosling O., Morgan-Hughes G., Iyengar S., Strain W., Loader R., Shore A., Roobottom C. (2013). Computed tomography to diagnose coronary artery disease: A reduction in radiation dose increases applicability. Clin. Radiol..

[15] Zenooz N.A., Zahka K.G., Siwik E.S., Gilkeson R.C. (2010). Noncompaction syndrome of the myocardium: Pathophysiology and imaging pearls. J. Thorac. Imaging.

[16] Rajiah P.S., Kalisz K., Broncano J., Goerne H., Collins J.D., Francois C.J., Ibrahim E.S., Agarwal P.P. (2022). Myocardial Strain Evaluation with Cardiovascular MRI: Physics, Principles, and Clinical Applications. Radiographics.

[17] Kalam K., Otahal P., Marwick T.H. (2014). Prognostic implications of global LV dysfunction: A systematic review and meta-analysis of global longitudinal strain and ejection fraction. Heart.

[18] Jacob S., Walker V., Fondard O., Chevelle C., Jimenez G., Bernier M.O., Laurier D., Ferrières J., Lairez O. (2019). Use of myocardial strain imaging by echocardiography for the early detection of radiotherapy-induced cardiotoxicity in breast cancer patients (BACCARAT Study). Arch. Cardiovasc. Dis. Suppl..

[19] Tang H.S., Kwan C.T., He J., Ng P.P., Hai S.H.J., Kwok F.Y.J., Sze H.F., So M.H., Lo H.Y., Fong H.T.A. (2023). Prognostic Utility of Cardiac MRI Myocardial Strain Parameters in Patients With Ischemic and Nonischemic Dilated Cardiomyopathy: A Multicenter Study. AJR Am. J. Roentgenol..

[20] Romano S., Judd R.M., Kim R.J., Kim H.W., Klem I., Heitner J.F., Shah D.J., Jue J., White B.E., Indorkar R. (2018). Feature-Tracking Global Longitudinal Strain Predicts Death in a Multicenter Population of Patients With Ischemic and Nonischemic Dilated Cardiomyopathy Incremental to Ejection Fraction and Late Gadolinium Enhancement. JACC Cardiovasc. Imaging.

[21] Kusunose K., Agarwal S., Marwick T.H., Griffin B.P., Popovic Z.B. (2014). Decision making in asymptomatic aortic regurgitation in the era of guidelines: Incremental values of resting and exercise cardiac dysfunction. Circ. Cardiovasc. Imaging.

[22] Li N., Zhang L., Wu H., Liu J., Cao Y., Li Y., Yu J., Han X., Shao G., Yang M. (2023). Quantifying left ventricular myocardial strain in patients with different CAD-RADS levels based on computed tomography feature tracking technology. Sci. Rep..

[23] Ahn Y., Koo H.J., Lee S.A., Jung D., Kang J.W., Yang D.H. (2024). Reference ranges of computed tomography-derived strains in four cardiac chambers. PLoS ONE.

[24] Messroghli D.R., Moon J.C., Ferreira V.M., Grosse-Wortmann L., He T., Kellman P., Mascherbauer J., Nezafat R., Salerno M., Schelbert E.B. (2017). Clinical recommendations for cardiovascular magnetic resonance mapping of T1, T2, T2* and extracellular volume: A consensus statement by the Society for Cardiovascular Magnetic Resonance (SCMR) endorsed by the European Association for Cardiovascular Imaging (EACVI). J. Cardiovasc. Magn. Reson..

[25] Zhuang B., Sirajuddin A., Wang S., Arai A., Zhao S., Lu M. (2018). Prognostic value of T1 mapping and extracellular volume fraction in cardiovascular disease: A systematic review and meta-analysis. Heart Fail. Rev..

[26] Ghostine S., Caussin C., Habis M., Habib Y., Clement C., Sigal-Cinqualbre A., Angel C.Y., Lancelin B., Capderou A., Paul J.F. (2008). Non-invasive diagnosis of ischaemic heart failure using 64-slice computed tomography. Eur. Heart J..

[27] Oda S., Emoto T., Nakaura T., Kidoh M., Utsunomiya D., Funama Y., Nagayama Y., Takashio S., Ueda M., Yamashita T. (2019). Myocardial Late Iodine Enhancement and Extracellular Volume Quantification with Dual-Layer Spectral Detector Dual-Energy Cardiac CT. Radiol. Cardiothorac. Imaging.

[28] Nishii T., Kobayashi T., Tanaka H., Kotoku A., Ohta Y., Morita Y., Umehara K., Ota J., Horinouchi H., Ishida T. (2022). Deep Learning-based Post Hoc CT Denoising for Myocardial Delayed Enhancement. Radiology.

[29] Abadia A.F., Aquino G.J., Schoepf U.J., Wels M., Schmidt B., Sahbaee P., Dargis D.M., Burt J.R., Varga-Szemes A., Emrich T. (2022). Automated Dual-energy Computed Tomography-based Extracellular Volume Estimation for Myocardial Characterization in Patients With Ischemic and Nonischemic Cardiomyopathy. J. Thorac. Imaging.

[30] le Polain de Waroux J.B., Pouleur A.C., Goffinet C., Pasquet A., Vanoverschelde J.L., Gerber B.L. (2008). Combined coronary and late-enhanced multidetector-computed tomography for delineation of the etiology of left ventricular dysfunction: Comparison with coronary angiography and contrast-enhanced cardiac magnetic resonance imaging. Eur. Heart J..

[31] Bandula S., White S.K., Flett A.S., Lawrence D., Pugliese F., Ashworth M.T., Punwani S., Taylor S.A., Moon J.C. (2013). Measurement of myocardial extracellular volume fraction by using equilibrium contrast-enhanced CT: Validation against histologic findings. Radiology.

[32] Ohta Y., Kishimoto J., Kitao S., Yunaga H., Mukai-Yatagai N., Fujii S., Yamamoto K., Fukuda T., Ogawa T. (2020). Investigation of myocardial extracellular volume fraction in heart failure patients using iodine map with rapid-kV switching dual-energy CT: Segmental comparison with MRI T1 mapping. J. Cardiovasc. Comput. Tomogr..

[33] Yashima S., Takaoka H., Iwahana T., Nishikawa Y., Ota J., Aoki S., Kinoshita M., Takahashi M., Sasaki H., Suzuki-Eguchi N. (2023). Evaluation of extracellular volume by computed tomography is useful for prediction of prognosis in dilated cardiomyopathy. Heart Vessel..

[34] Taylor A.J., Cerqueira M., Hodgson J.M., Mark D., Min J., O’Gara P., Rubin G.D., American College of Cardiology Foundation Appropriate Use Criteria Task Force, Society of Cardiovascular Computed Tomography, American College of Radiology (2010). ACCF/SCCT/ACR/AHA/ASE/ASNC/NASCI/SCAI/SCMR 2010 Appropriate Use Criteria for Cardiac Computed Tomography. A Report of the American College of Cardiology Foundation Appropriate Use Criteria Task Force, the Society of Cardiovascular Computed Tomography, the American College of Radiology, the American Heart Association, the American Society of Echocardiography, the American Society of Nuclear Cardiology, the North American Society for Cardiovascular Imaging, the Society for Cardiovascular Angiography and Interventions, and the Society for Cardiovascular Magnetic Resonance. Circulation.

[35] Budoff M.J., Dowe D., Jollis J.G., Gitter M., Sutherland J., Halamert E., Scherer M., Bellinger R., Martin A., Benton R. (2008). Diagnostic performance of 64-multidetector row coronary computed tomographic angiography for evaluation of coronary artery stenosis in individuals without known coronary artery disease: Results from the prospective multicenter ACCURACY (Assessment by Coronary Computed Tomographic Angiography of Individuals Undergoing Invasive Coronary Angiography) trial. J. Am. Coll. Cardiol..

[36] Ergun E., Kosar P., Ozturk C., Basbay E., Koc F., Kosar U. (2011). Prevalence and extent of coronary artery disease determined by 64-slice CTA in patients with zero coronary calcium score. Int. J. Cardiovasc. Imaging.

[37] Cury R.C., Leipsic J., Abbara S., Achenbach S., Berman D., Bittencourt M., Budoff M., Chinnaiyan K., Choi A.D., Ghoshhajra B. (2022). CAD-RADS 2.0—2022 Coronary Artery Disease—Reporting and Data System An Expert Consensus Document of the Society of Cardiovascular Computed Tomography (SCCT), the American College of Cardiology (ACC), the American College of Radiology (ACR) and the North America Society of Cardiovascular Imaging (NASCI). Radiol. Cardiothorac. Imaging.

[38] Vliegenthart R., Henzler T., Moscariello A., Ruzsics B., Bastarrika G., Oudkerk M., Schoepf U.J. (2012). CT of coronary heart disease: Part 1, CT of myocardial infarction, ischemia, and viability. AJR Am. J. Roentgenol..

[39] Nagao M., Matsuoka H., Kawakami H., Higashino H., Mochizuki T., Murase K., Uemura M. (2008). Quantification of myocardial perfusion by contrast-enhanced 64-MDCT: Characterization of ischemic myocardium. AJR Am. J. Roentgenol..

[40] Gopalan D., Raj V., Hoey E.T. (2010). Cardiac CT: Non-coronary applications. Postgrad. Med. J..

[41] Bhasin D., Sharma A., Sharma Y.P., Singhal M. (2024). Utility of Cardiac Computed Tomography in the Diagnosis of Apical Hypertrophic Cardiomyopathy: A Case Series. Heart Views.

[42] Jan M.F., Tajik A.J. (2017). Modern Imaging Techniques in Cardiomyopathies. Circ. Res..

[43] Dell’Aversana S., Ascione R., De Giorgi M., De Lucia D.R., Cuocolo R., Boccalatte M., Sibilio G., Napolitano G., Muscogiuri G., Sironi S. (2022). Dual-Energy CT of the Heart: A Review. J. Imaging.

[44] Azevedo O., Cordeiro F., Gago M.F., Miltenberger-Miltenyi G., Ferreira C., Sousa N., Cunha D. (2021). Fabry Disease and the Heart: A Comprehensive Review. Int. J. Mol. Sci..

[45] Venlet J., Tao Q., de Graaf M.A., Glashan C.A., de Riva Silva M., van der Geest R.J., Scholte A.J., Piers S.R.D., Zeppenfeld K. (2020). RV Tissue Heterogeneity on CT: A Novel Tool to Identify the VT Substrate in ARVC. JACC Clin. Electrophysiol..

[46] Maffei E., Messalli G., Martini C., Nieman K., Catalano O., Rossi A., Seitun S., Guaricci A.I., Tedeschi C., Mollet N.R. (2012). Left and right ventricle assessment with Cardiac CT: Validation study vs. Cardiac MR. Eur. Radiol..

[47] Muser D., Nucifora G., Castro S.A., Enriquez A., Chahal C.A.A., Magnani S., Kumareswaran R., Arkles J., Supple G., Schaller R. (2021). Myocardial Substrate Characterization by CMR T_1_ Mapping in Patients With NICM and No LGE Undergoing Catheter Ablation of VT. JACC Clin. Electrophysiol..

[48] Conte E., Mushtaq S., Carbucicchio C., Piperno G., Catto V., Mancini M.E., Formenti A., Annoni A., Guglielmo M., Baggiano A. (2021). State of the art paper: Cardiovascular CT for planning ventricular tachycardia ablation procedures. J. Cardiovasc. Comput. Tomogr..

